# Atomistic Descriptors for Machine Learning Models of Solubility Parameters for Small Molecules and Polymers

**DOI:** 10.3390/polym14010026

**Published:** 2021-12-22

**Authors:** Mingzhe Chi, Rihab Gargouri, Tim Schrader, Kamel Damak, Ramzi Maâlej, Marek Sierka

**Affiliations:** 1 Otto Schott Institute of Materials Research, Friedrich Schiller University Jena, 07743 Jena, Germany; mingzhe.chi@uni-jena.de (M.C.); tim.schrader@uni-jena.de (T.S.); 2 Georesources Materials Environment and Global Changes Laboratory (GEOGLOB), Faculty of Sciences of Sfax, Sfax University, Sfax 3018, Tunisia; rihab.gargouri.etud@fss.usf.tn (R.G.); kamel.damak@fss.usf.tn (K.D.); ramzi.maalej@fss.usf.tn (R.M.)

**Keywords:** machine learning, polymer, properties prediction

## Abstract

Descriptors derived from atomic structure and quantum chemical calculations for small molecules representing polymer repeat elements were evaluated for machine learning models to predict the Hildebrand solubility parameters of the corresponding polymers. Since reliable cohesive energy density data and solubility parameters for polymers are difficult to obtain, the experimental heat of vaporization $\Delta H_{\mathrm{vap}}$ of a set of small molecules was used as a proxy property to evaluate the descriptors. Using the atomistic descriptors, the multilinear regression model showed good accuracy in predicting $\Delta H_{\mathrm{vap}}$ of the small-molecule set, with a mean absolute error of 2.63 kJ/mol for training and 3.61 kJ/mol for cross-validation. Kernel ridge regression showed similar performance for the small-molecule training set but slightly worse accuracy for the prediction of $\Delta H_{\mathrm{vap}}$ of molecules representing repeating polymer elements. The Hildebrand solubility parameters of the polymers derived from the atomistic descriptors of the repeating polymer elements showed good correlation with values from the CROW polymer database.

## 1. Introduction

Computer-aided predictions of polymer solubility and miscibility with small molecules and drugs are of fundamental importance in a number of industrial applications, including the use of polymers as drug carriers in the growing field of nanomedicine [1]. Among various approaches, solubility and miscibility predictions based on Hildebrand solubility parameters are often used for polymer blends, polymer solutions and polymer–drug mixtures [2]. The Hildebrand model uses a solubility parameter (SP), δ, defined as the square root of the cohesive energy density:

(1) $$\delta =\sqrt{\frac{E_{\mathrm{coh}}}{V_{m}}}$$

where $E_{\mathrm{coh}}$ is the cohesive energy, and $V_{m}$ is the molar volume. The miscibility of two substances can be estimated by comparing the absolute value of the difference in their SPs. If it is more than 2 MPa^1/2^, the two substances are deemed immiscible, and with a difference of less than 2 MPa^1/2^, they are considered miscible [3]. The factor 2 MPa^1/2^ was determined on the basis of empirical considerations [3]. The Hildebrand SP can also be used to roughly estimate the Flory–Huggins interaction parameter [4], which is another useful tool for predicting the miscibility of polymer blends [5].

For low-molecular-weight compounds, $E_{\mathrm{coh}}$ and δ can be estimated from the heat of vaporization:

(2) $$E_{\mathrm{coh}}\approx \Delta H_{\mathrm{vap}}-RT$$

where $\Delta H_{\mathrm{vap}}$ is the heat of vaporization [2]. However, for polymers, SP is difficult to obtain from experiments [6]. Various experimental methods can be used to indirectly derive SP, such as hot-stage microscopy, differential scanning calorimetry (DSC) and ultraviolet spectroscopy [7,8], but these methods provide limited accuracy and can only be used for a small range of polymer species.

In addition to experimental methods, SP can also be calculated by empirical approaches and computer simulations. A group-contribution method (GC) is an empirical approach, which uses the sum of the contributions of structural and functional groups to estimate polymer properties [9]. GC is easy to apply but has limited accuracy due to the use of empirical assumptions. Although new GC approaches are being developed, a general model that can cover a wide range of polymer species and polymer properties is not available [10]. Atomistic simulations employing force fields and interatomic potential functions are another tool for predicting polymer properties [5,11]. However, accurate SP predictions using atomistic simulations are computationally demanding, especially for polymers and compounds with complex structures [12].

**Figure 1:**
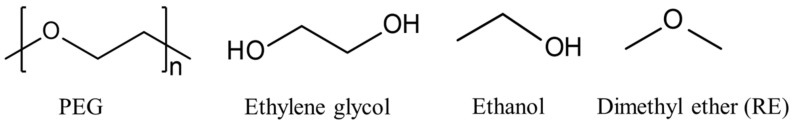
Polyethylene glycol (PEG) and different choices of small molecules with a structural motif of the repeating element of PEG.

In this regard, data-driven approaches based on machine learning (ML) models have become an appealing alternative to simple empirical approaches and atomistic simulations. ML models have been developed to predict the physical or chemical properties of materials with good accuracy, including solubility parameters [11]. However, the predictive power of ML models depends heavily on the availability of accurate and consistent target data covering a wide structural and compositional range, as well as unbiased descriptors, i.e., readily available observables that can be linked to the target property [13]. Such observables can be derived, e.g., from experimental data or from quantum chemical or atomistic calculations [13,14]. It is difficult to obtain relevant experimental data on target properties and descriptors for predicting the SP of polymers. In the case of descriptors, one approach is to use the features derived for monomer molecules [13]. However, polymer properties may be fundamentally different from those of the monomer. Therefore, small organic molecules that are structurally similar to the repeating element (RE) of the polymer may be a better choice. For a given polymer, different molecules can be identified that represent REs, as shown in Figure 1 for polyethylene glycol (PEG). Both ethylene glycol and ethanol, as polar molecules forming strong hydrogen bonds, are poor choices for deriving molecular descriptors for ML models for PEG.

In this work, descriptors derived from the atomic structure and quantum chemical calculations of small molecules as potential polymer REs are evaluated for ML models of the polymer SP. Since reliable cohesive energy density and SP data for polymers are difficult to obtain in experiments and simulations, a surrogate target property is used to evaluate the descriptors, namely, the experimental heat of vaporization $\Delta H_{\mathrm{vap}}$ of the small molecules. For low-molecular-weight compounds, $\Delta H_{\mathrm{vap}}$ can usually be determined with good accuracy [15]. Subsequently, the relationship between $\Delta H_{\mathrm{vap}}$ of the polymer RE and the available SP of the polymers is investigated.

## 2. Method

### 2.1. Molecular Datasets

**Figure 2:**
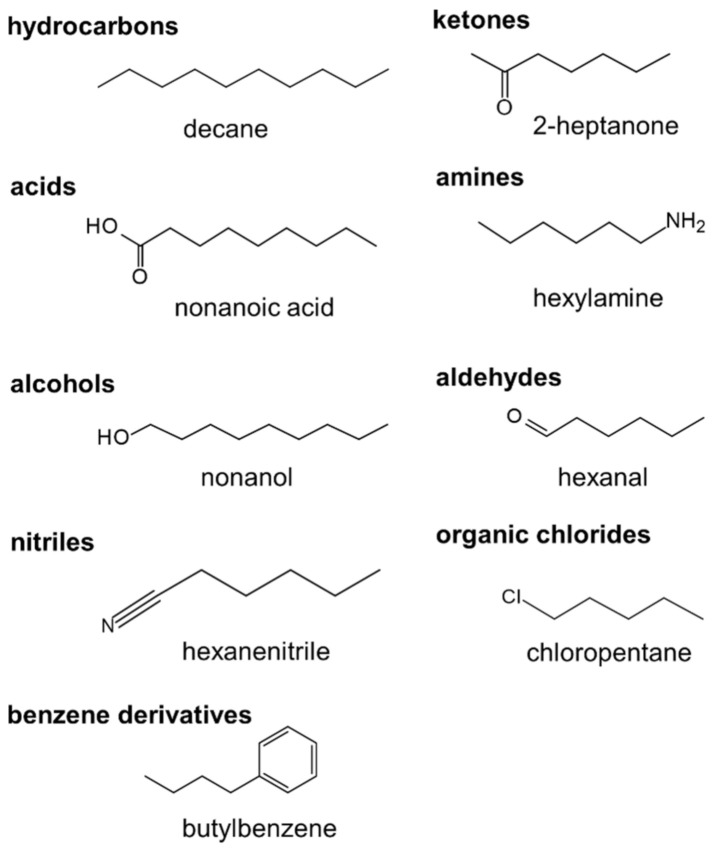
The largest molecule of each type used in the training dataset: decane (C_10_H_22_), nonanoic acid (C_8_H_17_COOH), nonanol (C_9_H_19_OH), 2-heptanone (C_7_H_14_O), hexylamine (C_6_H_13_NH_2_), hexanal (C_5_H_11_CHO), hexanenitrile (C_5_H_11_CN), chloropentane (C_5_H_11_Cl) and butylbenzene (C_6_H_5_C_4_H_9_).

ML models for predicting $\Delta H_{\mathrm{vap}}$ were trained and tested on a dataset of small organic molecules including hydrocarbons, alcohols, acids, amines, ketones, aldehydes, nitriles, organic chlorides and benzene derivatives. A summary of the dataset is shown in Table 1. Figure 2 shows examples of the largest molecules used. The ML models were then applied to another dataset of organic molecules with structural similarity to REs of popular polymers to predict $\Delta H_{\mathrm{vap}}$ and correlate it with the polymer SP. This dataset is summarized in Table 2.

The organic molecules in both datasets cover a wide range of $\Delta H_{\mathrm{vap}}$ values, from 8.19 kJ/mol (methane) to 69.00 kJ/mol (heptanoic acid), and represent different chemical structures. The experimental values of $\Delta H_{\mathrm{vap}}$ for all molecules, measured around a normal boiling point, were collected from the literature (see Supplementary Materials).

### 2.2. Computational Details

All density functional theory calculations were performed as a gas phase using the Turbomole program package [16]. The Becke 3-parameter Lee–Yang–Parr (B3-LYP) [17] exchange–correlation functional was employed, along with triple zeta valence plus polarization (def2-TZVP) [18] basis sets and Grimme dispersion correction (DFT-D3) [19]. The geometry convergence criteria for DFT calculations were $10^{-6}$ hartree for the energy change and $10^{-3}$ hartree/bohr for the gradient norm. Count descriptors and topological descriptors were calculated with the PaDEL-Descriptor program [20]. Python 3 with the Scikit-learn package was used for building all machine learning models [21].

### 2.3. Molecular Descriptors

In the current work, four quantum chemical descriptors were obtained using DFT calculations: atomization energy (AE), quadrupole moment (QM), chemical hardness η and electronegativity χ. There are different definitions for the quadrupole moment [22,23]. In the present work, the quadrupole moment was defined as the second moment of charge [23], and QM was taken as

(3) $$\mathrm{QM}=\frac{1}{3}\left(Q_{\mathrm{xx}}+Q_{\mathrm{yy}}+Q_{\mathrm{zz}}\right)$$

where $Q_{\mathrm{xx}}$, $Q_{\mathrm{yy}}$ and $Q_{\mathrm{zz}}$ are diagonal elements of the second moment of the charge tensor.

Chemical hardness η and electronegativity χ are chemical reactivity descriptors that were applied in an artificial neural network for predicting solvation energies [24]. They are defined as

(4) $$\eta ≃E_{\mathrm{LUMO}}-E_{\mathrm{HOMO}}$$

and

(5) $$\chi =-\frac{1}{2}\left(E_{\mathrm{LUMO}}+E_{\mathrm{HOMO}}\right)$$

where $E_{\mathrm{HOMO}}$ and $E_{\mathrm{LUMO}}$ denote the energies of the highest occupied (HOMO) and lowest unoccupied molecular orbitals (LUMO), respectively.

The remaining descriptors, such as number of aromatic bonds (nAromBond) and number of heavy atoms (nHeavyAtom), were generated using the PaDEL-Descriptor [20] program based on the atomic structures of molecules. The descriptors were obtained using exactly the same method for both datasets (Table 1 and Table 2). The full specification of the descriptors is given in the Supplementary Materials.

### 2.4. Machine Learning Models

Two supervised machine learning models were used: multilinear regression (MLR) and kernel ridge regression (KRR) [25]. MLR is the simplest ML model using the least square method and has been widely applied in data analysis. The MLR model can be represented as a linear combination of all descriptors

(6) $$y_{\mathrm{prediction}}=\theta_{1}x_{1}+\theta_{2}x_{2}+\cdots +\theta_{i}x_{i}+\theta_{0}\;$$

where $\theta_{i}$ is the coefficient of each descriptor $x_{i}$, and $\theta_{0}$ is the intercept. The training of the MLR model involves the determination of the best $\left(\theta_{1},\theta_{2},\ldots ,\theta_{i}\right)$ and $\theta_{0}$. All hyperparameters used for MLR training used the default values implemented in the Scikit-learn package.

KRR is a combination of the kernel function and ridge regression, which is an improvement on the ordinary linear regression method [26,27]. There are several kernel functions available for different tasks, and in the present work, the polynomial kernel function was applied

(7) $$k\left(x,x^{\prime }\right)={\left(x\cdot x^{\prime }+c\right)}^{d},$$

where x  and $x^{’\;}$ are descriptors, and hyperparameters *c* and *d* are the soft margin constant and degree of the polynomial kernel, respectively. The accuracy and performance of the model usually depend on the choice of hyperparameters. Since the dataset was relatively small, changes in parameters other than *c* and *d* had little effect on the model accuracy and were therefore set to constant values (alpha (regularization strength) = 0.001 and gamma = none). In the current work, *c* and *d* in Equation (7) were determined using the grid search function of Scikit-learn. Based on the grid search results, the value of *c* had little effect on the model and was finally set to 1. The models with *d* = 1 and *d* = 2 showed similar performance, and both models were retained for further study. Changes in parameters other than *c* and *d* had little effect on the model accuracy and were therefore left at the default values implemented in the Scikit-learn package.

Due to the limited size of the dataset (61 molecules in total), the leave-one-out cross-validation (LOOCV) method was used in the current work, aiming to make optimal use of each sample and to obtain a more justified model. LOOCV is an extreme case of cross-validation, in which only one sample is selected for testing in each cycle, and the other samples are used to train the model until all samples have been selected once. The final model is optimized by averaging the LOOCV results. MLR and KRR models were trained with the same dataset, and LOOCV was applied for all models. After training and LOOCV, all models were used to predict $\Delta H_{\mathrm{vap}}$ of polymer REs (16 molecules in Table 2), and the performance of all models was analyzed [28] using root-mean-square error (RMSE)

(8) $$\mathrm{RMSE}=\sqrt{\frac{1}{n}\sum_{i=1}^{n}{\left(y_{i}-{\hat{y}}_{i}\right)}^{2}}$$

mean absolute error (MAE)

(9) $$\mathrm{MAE}=\frac{1}{n}\sum_{i=1}^{n}\left|y_{i}-{\hat{y}}_{i}\right|$$

and the average of relative error (ARE)

(10) $$\mathrm{ARE}=\frac{1}{n}\sum_{i=1}^{n}\left|1-\frac{{\hat{y}}_{i}}{y_{i}}\right|\;$$

where $y_{i}$ and ${\hat{y}}_{i}$ are the reference and predicted values, respectively. In addition, the coefficient of determination (R^2^) was used to describe the proportion of variability in a dataset that can be explained by the model [29].

## 3. Discussion

### 3.1. Selection of Molecular Descriptors

**Figure 3:**
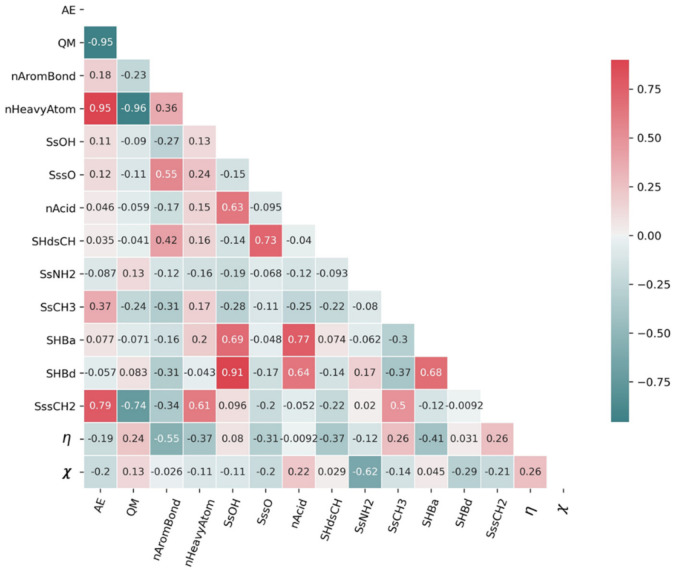
Correlation coefficients among 15 descriptors. AE: atomization energy, QM: quadrupole moment, nAromBond: number of aromatic bonds; nHeavyAtom: number of heavy atoms (all but hydrogen); SsOH: sum of (-OH) E-States; SssO: sum of (-O-) E-States; nAcid: number of acidic groups; SHdsCH: sum of (=CH-) E-States; SsNH2: sum of (-NH2) E-States; SsCH3: sum of (-CH3) E-States; SHBa: sum of E-States for hydrogen bond acceptors; SHBd: sum of E-States for hydrogen bond donors; SssCH2: sum of (-CH2) E-States (see Supplementary Materials); η: chemical hardness; χ: electronegativity.

Molecular descriptors were manually selected and filtered by analyzing multicollinearity based on correlation coefficients. For this, descriptors were selected in addition to the quantum chemical descriptors that showed low multicollinearity (correlation coefficient within ±0.75) and a high degree of correlation with $\Delta H_{\mathrm{vap}}$. The 15 descriptors finally selected are shown in Figure 3.

Figure 3 shows that there is only weak correlation among most descriptors, which can reduce the risk of collinearity problems [30]. Reducing redundant and irrelevant descriptors also lowers the cost of training and reduces the possibility of an overfitting problem [14,31].

### 3.2. Predictions of $\Delta H_{\mathrm{vap}}$ for Small Organic Molecules

The final MLR model for predicting $\Delta H_{\mathrm{vap}}$ (in kJ/mol) is given as

(11) $$\begin{array}{c} \Delta H_{\mathrm{vap}}=-23.723\mathrm{AE}+0.234\mathrm{QM}-3.303\mathrm{nAromBond}+3.601\mathrm{SsOH}-0.33\mathrm{SssO}+0.477\mathrm{SsCH}3+2.753\mathrm{SsNH}2\\ \;-0.65\mathrm{SHBa}+1.301\mathrm{SHdsCH}-5.580\mathrm{nAcid}-0.618\mathrm{SssCH}2-15.805\mathrm{SHBd}\\ \;+15.53\mathrm{nHeavyAtom}+10.667 \end{array}$$

**Figure 4:**
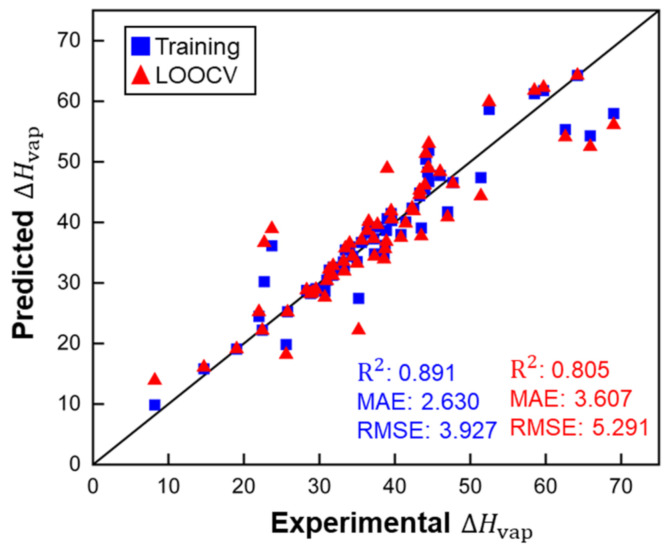
Performance of the MLR model for $\Delta H_{\mathrm{vap}}$ predictions of small molecules (ARE for the training set: 0.072 and for LOOCV: 0.105). $\Delta H_{\mathrm{vap}}$, MAE and RMSE in kJ/mol.

This model does not contain any unreasonably small or large factors for descriptors, which indicates that there are no irrelevant or redundant descriptors. Figure 4 shows that MLR performed well for the training set of small molecules and the LOOCV, according to the R^2^ score and other metrics. ARE for training (0.071) and LOOCV (0.105) showed the same trend as the other metrics. The MLR model showed good accuracy for predicting $\Delta H_{\mathrm{vap}}$ of molecules, with a maximum deviation of 12.43 kJ/mol for ethanoic acid. However, there are large disparities in the values of $\Delta H_{\mathrm{vap}}\;$ for ethanoic acid across the literature (from 23.7 (at 391.1 K) to 42 (at 305 K) kJ/mol) [32,33]. The overall deviation is within experimental accuracy. For the LOOCV, both the RMSE of 5.291 kJ/mol and MAE of 3.607 kJ/mol are within ranges indicative of good accuracy.

**Figure 5:**
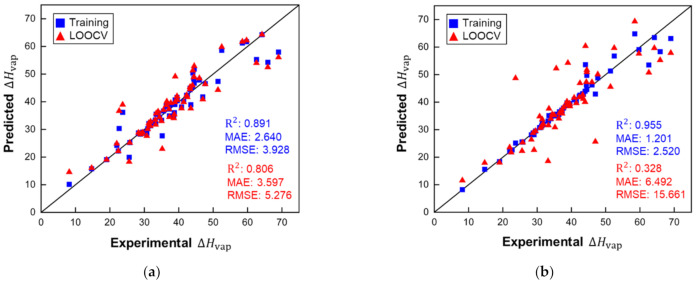
Performance of the KRR models for $\Delta H_{\mathrm{vap}}$ predictions of small molecules: (**a**) *d* = 1 (ARE for the training set: 0.072 and for LOOCV: 0.106), (**b**) *d* = 2 (ARE for the training set: 0.026 and for LOOCV: 0.184). $\Delta H_{\mathrm{vap}}$, MAE and RMSE in kJ/mol.

The performance comparison of the final KRR (*d* = 1) and KRR (*d* = 2) models is shown in Figure 5.

Compared to the MLR model, the KRR model (*d* = 1) did not perform better in training, but all metrics had a small lead in cross-validation, which showed slightly better stability. KRR (*d* = 2) performed best during training but was the worst in LOOCV, and this case was most likely due to the overfitting. Considering the size of the datasets used in the current work, high-scoring ML models trained with small datasets can often suffer from overfitting.

### 3.3. Predictions of $\Delta H_{vap}$ for Polymer Repeating Elements

**Figure 6:**
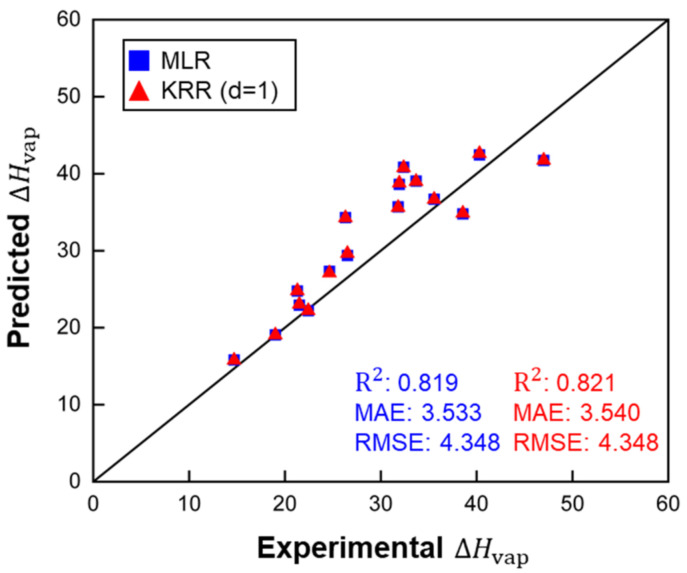
$\Delta H_{\mathrm{vap}}$ predictions of polymer RE by MLR and KRR (*d* = 1), ARE for MLR: 0.118 and for KRR (*d* = 1): 0.118. $\Delta H_{\mathrm{vap}}$, MAE and RMSE in kJ/mol.

All three models were applied to predict $\Delta H_{\mathrm{vap}}$ of molecules representing polymer REs. Table 3 and Figure 6 show that the MLR and KRR (*d* = 1) models provided the best accuracy. The KRR (*d* = 2) model failed to predict $\Delta H_{\mathrm{vap}}$ of polymer RE, and the much larger error of the KRR (*d* = 2) suggests that the model was overfitted. As mentioned, KRR algorithms do not offer advantages on small datasets.

Figure 4, Figure 5 and Figure 6 demonstrate that the MLR model showed slightly worse performance than the two KRR models during training and cross-validation, but the MAE of MLR for polymer RE was better than that of KRR (*d* = 1). Therefore, the MLR model and the KRR model (*d* = 1) in the current work have better extrapolation ability than the KRR (*d* = 2) models. However, the KRR algorithm with higher *d* could still yield better results for a larger dataset with more complex structures and chemical compositions.

### 3.4. Hildebrand Solubility Parameter of Polymers

**Figure 7:**
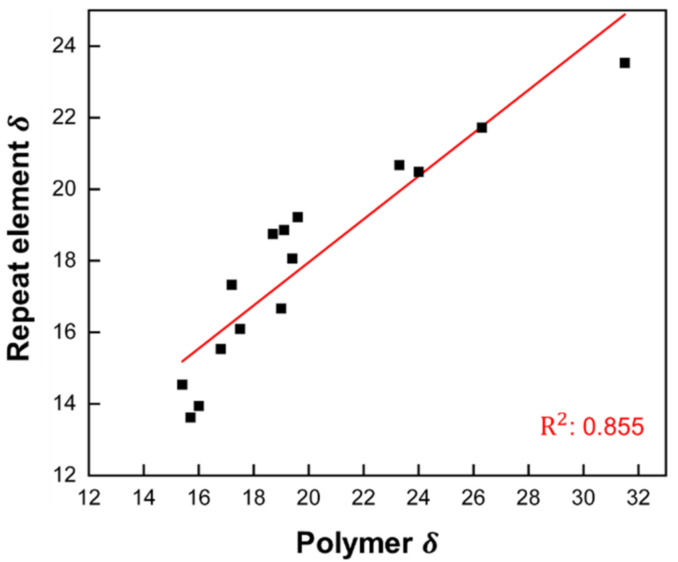
Correlation of Hildebrandt SP between polymers and REs. All values in MPa^1/2^. Linear fit model: $\delta_{\mathrm{polymer}}=0.602\delta_{\mathrm{RE}}+5.915$.

Our results show that the heat of vaporization of small molecules and polymeric REs, and thus their SPs, can be predicted with good accuracy using the MLR and KRR (*d* = 1) models. The question now is how well the Hildebrand SP of RE correlates with the SP of the corresponding polymers. For this, Hildebrand SPs of polymers were collected from the CROW polymer database [34] with recommended values, and SPs of REs were calculated from MLR-predicted $\Delta H_{\mathrm{vap}}$ (see Supplementary Materials). Figure 7 shows the correlation of Hildebrand SPs between polymers and REs. The linear model yields an R^2^ value of 0.855.

There are several factors that can affect the accuracy of Hildebrand SP predictions for polymers. First, the experimental values of SPs for polymers can only be determined indirectly, and the accuracy of such values is essentially indeterminate. Second, the SPs are determined not only by the internal structure of the polymer chains, reflected here in the descriptors derived from the polymer RE, but also by factors such as the degree of polymerization, polydispersity, and the nature of the end groups. Such factors cannot be determined from the properties of REs alone and must be derived from experimental data. How well the two descriptors, chemical hardness and electronegativity, actually help in the prediction of solubility parameters needs to be further investigated, as intuitively, the association between the two and polymer solubility is not strong. In addition, larger chemical structures, such as oligomers with several repeating units, may provide more information about inter- and intramolecular interactions and can improve the accuracy of machine learning models. Simulations of such structures are obviously less computationally expensive than simulations of polymers, but finding suitable descriptors may still be a challenge. This is also one of the pathways for future research studies.

## 4. Conclusions

In this work, descriptors derived from atomic structure and quantum chemical calculations for small molecules as potential polymer repeating elements were evaluated for machine learning models to predict the Hildebrand solubility parameters of the corresponding polymers. Since reliable cohesive energy density data and solubility parameters for polymers are difficult to obtain, the experimental heat of vaporization $\Delta H_{\mathrm{vap}}$ of small molecules was used as a proxy property to evaluate the descriptors. The multilinear and kernel ridge regression model (with polynomial kernel degree = 1) showed good and very similar performance in training, cross-validation and the prediction of molecules representing polymer repeating elements. The kernel ridge regression model (degree = 2) was strongly overfitted, which was revealed by its poor performance in cross-validation and prediction. The Hildebrand solubility parameters derived from the multilinear regression model for the $\Delta H_{\mathrm{vap}}$ of polymer repeating elements showed good correlation with the solubility parameters of the corresponding polymers collected from the CROW polymer database. However, atomistic descriptors derived from polymer repeating elements only reflect the internal structure of the polymer chains. More accurate models for predicting the Hildebrand solubility parameters of polymers must take into account additional relevant factors, such as the degree of polymerization, polydispersity and the nature of the polymer end groups. Such factors cannot be determined from the properties of the repeating elements of the polymer alone and must be derived from experimental data.

## Figures and Tables

**Table 1:** Summary of the molecules included in the training dataset with experimental $\Delta H_{\mathrm{vap}}$ (see Supplementary Materials, Tables S1 and S6).

Type	Formula	Size *n*	Number of Molecules
hydrocarbons	C_n_H_2n+2_	1–10	10
acids	C_n_H_2n+1_COOH	0–8	9
alcohols	C_n_H_2n+1_OH	1–9	9
ketones	C_n_H_2n_O/C_6_H_5_COCH_3_	3–7	6
amines	C_n_H_2n+1_NH_2_	1–6	5
aldehydes	C_n−1_H_2n−1_CHO/C_6_H_5_CHO	3–6	5
nitriles	C_n_H_2n+1_CN	1, 3–6	5
organic chlorides	C_n_H_2n+1_Cl	1, 3–6	5
benzene derivatives	C_6_H_6_/C_6_H_5_OCH_3_/C_6_H_5_OCH_2_CH_3_/C_6_H_5_CH_2_OCH_3_ /C_6_H_5_C_n_H_2n+1_	1, 2, 4	7

**Table 2:** Summary of polymer repeating elements (REs) included in the validation set with experimental $\Delta H_{\mathrm{vap}}$ (see Supplementary Materials, Tables S2 and S7).

Polymer	Formula	RE	Formula of RE
poly(acrylic acid)	(C_3_H_4_O2)_n_	propanoic acid	CH_3_CH_2_COOH
poly(allyl cyanide)	(C_4_H_5_N)_n_	butanenitrile	CH_3_CH_2_CH2CN
polyacrylonitrile	(C_3_H_3_N)_n_	propanenitrile	CH_3_CH_2_CN
polybutylene	(C_4_H_8_)_n_	butane	CH_3_CH_2_CH_2_CH_3_
polyethylene (HDPE)	(C_2_H_4_)_n_	ethane	CH_3_CH_3_
poly(ethylene glycol)	(C_2_H_4_O)_n_	dimethyl ether	CH_3_OCH_3_
*cis*-1,4-polyisoprene	(C_5_H_8_)_n_	2-methyl-2-butene	CH_3_CHC(CH_3_)_2_
polyisobutene	(C_4_H_8_)_n_	isobutane	(CH_3_)_2_CHCH_3_
polymethacrylonitrile	(C_4_H_5_N)_n_	isobutyronitrile	(CH_3_)_2_CHCN
poly(methyl methacrylate)	(C_5_H_8_O_2_)_n_	methyl butyrate	CH_3_CH_2_CH_2_COOCH_3_
polypropylene	(C_3_H_6_)_n_	propane	CH_3_CH_2_CH_3_
polystyrene	(C_8_H_8_)_n_	ethylbenzene	C_6_H_5_C_2_H_5_
poly(vinyl alcohol)	(C_2_H_4_O)_n_	ethanol	CH_3_CH_2_OH
poly(vinyl acetate)	(C_4_H_6_O2)_n_	ethyl acetate	CH_3_COOCH_2_CH_3_
poly(vinyl chloride)	(C_2_H_3_Cl)_n_	chloroethane	CH_3_CH_2_Cl
poly(vinyl ethyl ether)	(C_4_H_6_O)_n_	diethyl ether	CH_3_CH_2_OCH_2_CH_3_

**Table 3:** Performance comparison of MLR and KRR models (MAE and RMSE in kJ/mol).

	Training Set (LOOCV)				Polymer REs			
	R^2^	MAE	RMSE	ARE	R^2^	MAE	RMSE	ARE
MLR	0.805	3.607	5.291	0.105	0.819	3.533	4.348	0.118
KRR (d=1)	0.806	3.597	5.276	0.106	0.821	3.540	4.348	0.118
KRR (d=2)	0.328	2.520	15.661	0.184	0.311	23.472	36.536	0.820

## Data Availability

All related data and results can be found in Supplementary Materials.

## Supplementary Materials

The following are available online at https://www.mdpi.com/article/10.3390/polym14010026/s1, Table S1: Heat of vaporization $\Delta H_{\mathrm{vap}}$ of small molecules; Table S2: Heat of vaporization $\Delta H_{\mathrm{vap}}$ of polymer REs; Table S3: MLR predictions on polymer RE dataset; Table S4: KRR predictions on polymer RE dataset; Table S5: Hildebrand solubility parameter δ of polymers and calculated δ of REs; Table S6: Complete dataset of small organic molecules; Table S7: Complete dataset of polymer REs.
