# Supplementary material for: Atomistic Descriptors for Machine Learning Models of Solubility Parameters for Small Molecules and Polymers
Source: Polymers (Basel). 2021 Dec 22;14(1):26. doi: 10.3390/polym14010026 (PMC8747575; doi:10.3390/polym14010026)
Supplement: Supplementary file 1 [file polymers-14-00026-s001.zip › polymers-1463250-supplementary.pdf]

Supplementary Materials

Atomistic descriptors for machine learning models of solubility parameters for small molecules and polymers

Mingzhe Chi [1], Rihab Gargouri [2], Tim Schrader [1], Kamel Damak [2], Ramzi Maâlej [2] and Marek Sierka [1,*]

[1] Otto Schott Institute of Materials Research, Friedrich Schiller University Jena, 07743 Jena, Germany; mingzhe.chi@uni-jena.de (M.C.); tim.schrader@uni-jena.de (T.S.)
[2] Georesources Materials Environment and Global Changes Laboratory (GEOGLOB), Faculty of Sciences of Sfax, Sfax University, 3018 Sfax, Tunisia; rihab.gargouri.etud@fss.usf.tn (R.G.); kamel.damak@fss.usf.tn (K.D.); ramzi.maalej@fss.usf.tn (R.M.)
* Correspondence: marek.sierka@uni-jena.de

S1. Molecular descriptors

Descriptors derived from DFT calculations

AE: atomization energy,
QM: quadrupole moment.
η: chemical hardness
χ: electronegativity

Atom count descriptors

nAcid: Number of acidic groups.
nAtom: number of atoms,
nAromBond: Number of aromatic bonds
nHeavyAtom: Number of heavy atoms (i.e. not hydrogen)
nH: number of Hydrogen atom,
nO: number of Oxygen atom,
nN: number of Nitrogen atom,
nC: Number of carbon atom.

Bond count descriptors

nBonds: number of bonds,
nBonds2: total number of bonds (including bonds to hydrogens),
nBondsS: number of single bonds (including bonds with hydrogen),
nBondsS2: total number of single bonds (including bonds to hydrogens, excluding aromatic bonds),
nBondsS3: total number of single bonds (excluding bonds to hydrogens and aromatic bonds),
nBondsD: number of double bonds,
nBondsM: Total number of bonds that have bond order greater than one (aromatic bonds have bond order 1.5).

Group count descriptors

nsCH3: number of -CH3,
nssCH2: number of -CH2-,
ndsCH: number of =CH-,
ndssC: number of =C<,
ndO: number of =O,
nssO: number of -O-,
nsssCH: number of >CH-,
nsNH2: number of -NH2,
nsOH: number of -OH.

Property descriptors

MW: molecular weight,
AMW: average molecular weight,
apol: atomic polarizability.

Electrotopological State (E-state) Indices descriptors [1]

SHsOH: sum of (-OH) hydrogen E-States,
SHsNH2: sum of (-NH2) hydrogen E-States,
SdsCH: sum of (=CH-) E-States,
SdssC: sum of (=C<) E-States,
SdO: Sum of (=O) E-States.
SsOH: Sum of (-OH) E-States
SssO: Sum of (-O-) E-States
SHdsCH: Sum of (=CH-) E-States
SsNH2: Sum of (-NH2) E-States
SsCH3: Sum of (-CH3) E-States
SHBa: Sum of E-States for (strong) hydrogen bond acceptors
SHBd: Sum of E-States for (strong) hydrogen bond donors
SssCH2: Sum of (-CH2) E-State

S2 Data collection

Table S1. Experimental heat of vaporization ΔH_{vap} of small molecules used for training all machine learning models, ΔH_{vap} in kJ/mol

Molecule	ΔH_{vap}	Molecule	ΔH_{vap}
pentanal	37.3 [2]	ethane	14.69 [3]
methoxybenzene	38.97 [3]	nonane	37.18 [3]
octane	34.41 [3]	propanoic acid	47 [2]
2-hexanone	36.35 [3]	butylamine	31.81 [3]
hexanol	44.50 [3]	pentanol	44.36 [3]
butanal	31.5 [3]	pentane	25.79 [3]
propane	19.04 [3]	heptanone	39.5 [2]
butanol	43.29 [3]	ethanol	38.56 [3]
acetophenone	43.98 [3]	heptanol	62.6 [2]
toluene	33.18 [3]	heptanoic acid	69 [2]
propylamine	29.55 [3]	propanal	28.31 [3]
ethylbenzene	35.57 [3]	ethanoic acid	23.7 [3]
propanol	41.44 [3]	nonanoic acid	64.2 [2]
heptane	31.77 [3]	hexanal	40.8 [2]
nonanol	59.7 [2]	butanone	31.3 [3]
octanol	52.5 [2]	octanoic acid	58.5 [3]
acetone	29.1 [3]	2-pentanone	33.44 [3]
butane	22.44 [3]	methanol	35.21 [3]
hexane	28.85 [3]	butylbenzene	38.87 [3]
decane	39.58 [3]	methylamine	25.6 [3]
benzaldehyde	42.5 [3]	benzene	30.72 [3]
pentylamine	34.01 [3]	hexanoic acid	65.9 [2]
butanoic acid	47.7 [2]	benzyl methyl ether	51.4 [2]
hexylamine	36.54 [3]	phenetole	44.5 [2]
methane	8.19 [3]	chloromethane	22 [2]
pentanoic acid	44.1 [3]	chlorobuthane	35 [2]
methanoic acid	22.69 [3]	chloropentane	38.7 [2]
chlorohexane	43.5 [2]	butyronitrile	37.7 [2]
chloropropane	31 [2]	pentanenitrile	42.3 [2]
acetonitrile	33.3 [2]	hexanenitrile	43.3 [2]
heptanonitrile	46 [2]		

Table S2. Experimental heat of vaporization $\Delta H_{\mathrm{vap,RE}}$ of polymer repeating elements used for evaluating accuracy of machine learning models, $\Delta H_{\mathrm{vap,RE}}$ in kJ/mol

Polymer	Full name	RE	$\boldsymbol{\Delta H_{\mathrm{vap,RE}}}$
PAA	poly(acrylic acid)	propanoic acid	47 [4]
PAC	poly(allyl cyanide)	butanenitrile	33.68 [3]
PAN	polyacrylonitrile	propanenitrile	31.81 [3]
PB	polybutylene	butane	22.44 [3]
PE	polyethylene (HDPE)	ethane	14.69 [3]
PEG	polyethylene glycol	dimethyl ether	21.51 [3]
cis-PI	*cis*-1,4-polyisoprene	2-methyl-2-butene	26.31 [3]
PIB	polyisobutene	isobutane	21.30 [3]
PMAN	polymethacrylonitrile	isobutyronitrile	32.39 [3]
PMMA	polymethyl methacrylate	methyl butyrate	40.3 [2]
PP	polypropylene	propane	19 [3]
PS	polystyrene	ethylbenzene	35.57 [3]
PVA	polyvinyl alcohol	ethanol	38.56 [3]
PVAc	polyvinyl acetate	ethyl acetate	31.94 [3]
PVC	polyvinyl chloride	chloroethane	24.65 [3]
PVEE	polyvinyl ethyl ether	diethyl ether	26.52 [3]

Table S3. Heat of vaporization of polymer repeat units predicted by multi-linear regression model, $\Delta H_{\mathrm{vap,RE}}$ in kJ/mol

Polymer	RE	$\Delta H_{\mathrm{vap,RE}}$	Predicted $\Delta H_{\mathrm{vap,RE}}$	Relative error
PAA	propanoic acid	54.4	41.73	0.112
PAC	butanenitrile	33.68	39.04	0.159
PAN	propanenitrile	31.81	35.71	0.122
PB	butane	22.44	22.21	0.010
PE	ethane	14.69	15.81	0.076
PEG	dimethyl ether	21.51	22.93	0.066
cis-PI	2-methyl-2-butene	26.31	34.30	0.303
PIB	isobutane	21.30	24.77	0.163
PMAN	isobutyronitrile	32.39	40.85	0.261
PMMA	methyl butyrate	40.3	42.43	0.053
PP	propane	19	19.08	0.004
PS	ethylbenzene	35.57	36.67	0.031
PVA	ethanol	38.56	34.78	0.098
PVAc	ethyl acetate	31.94	38.62	0.209
PVC	chloroethane	24.65	27.32	0.108
PVEE	diethyl ether	26.52	29.39	0.108

Table S4. Heat of vaporization of polymer repeat units predicted by two kernel ridge regression models, $\Delta H_{\mathrm{vap,RE}}$ in kJ/mol

			Predicted $\Delta H_{\mathrm{vap,RE}}$			
**Polymer**	**RE**	$\Delta H_{\mathrm{vap,RE}}$	KRR (d = 1)	Relative error	KRR (d = 2)	Relative error
PAA	propanoic acid	54.40	41.74	0.112	42.94	0.086
PAC	butanenitrile	33.68	39.00	0.158	37.20	0.105
PAN	propanenitrile	31.81	35.62	0.120	34.05	0.070
PB	butane	22.44	22.21	0.010	22.00	0.020
PE	ethane	14.69	15.78	0.074	15.60	0.062
PEG	dimethyl ether	21.51	23.05	0.072	-25.21	2.172
cis-PI	2-methyl-2-butene	26.31	34.27	0.302	-23.91	1.909
PIB	isobutane	21.30	24.79	0.164	-13.75	1.646
PMAN	isobutyronitrile	32.39	40.78	0.259	7.61	0.765
PMMA	methyl butyrate	40.30	42.61	0.057	124.26	2.083
PP	propane	19.00	19.06	0.003	18.39	0.032
PS	ethylbenzene	35.57	36.66	0.031	35.63	0.002
PVA	ethanol	38.56	34.87	0.096	38.45	0.003
PVAc	ethyl acetate	31.94	38.77	0.214	106.06	2.321
PVC	chloroethane	24.65	27.10	0.099	26.17	0.062
PVEE	diethyl ether	26.52	29.63	0.117	-20.69	1.780

Table S5. Experimental hildebrand solubility parameter δ of polymers and calculated δ of polymer repeating elements, δ in $MPa^{1/2}$

Polymer	Polymer δ	Polymer repeating elements	Polymer repeating elements δ
PAA	21.3 [5]	propanoic acid	24.54
PAC	24 [6]	butanenitrile	17.82
PAN	26.3 [5]	propanenitrile	18.93
PB	16 [5]	butane	14
PE	16.8 [5]	ethane	16.1
PEG	19.4 [5]	dimethyl ether	17.26
cis-PI	17.2 [5]	2-methyl-2-butene	13.41
PIB	15.4 [5]	isobutane	13.76
PMAN	23.3 [5]	isobutyronitrile	17.8
PMMA	18.7 [5]	methyl butyrate	18.08
PP	15.7 [5]	propane	13.85
PS	19 [5]	ethylbenzene	16.55
PVA	31.5 [5]	ethanol	23.62
PVAc	19.6 [5]	ethyl acetate	18.95
PVC	19.1 [5]	chloroethane	20.97
PVEE	17.5 [5]	diethyl ether	14.77

Table S6. Descriptors of small organic molecules, AE: atomization energy, QM: quadrupole moment, nAromBond: number of aromatic bonds, nHeavyAtom: number of heavy atoms (all but hydrogen), SsOH: sum of (-OH) E-States, SssO: sum of (-O-) E-States, nAcid: number of acidic groups, SHdsCH: sum of (=CH-) E-States, SsNH2: sum of (-NH2) E-States, SsCH3: sum of (-CH3) E-States, SHBa: sum of E-States for hydrogen bond acceptors, SHBd: sum of E-States for hydrogen bond donors, SssCH2: sum of (-CH2) E-States (see Supplementary Materials), η: chemical hardness, χ: electronegativity

Molecule	AE	QM	nArom Bond	SsOH	SssO	SsCH3	SsNH2	SHBa	SHdsCH	nAcid	SssCH2	SHBd	nHeavy Atom	η	χ
pentanal	2.341	-31.039	0	0	0	2.073	0	9.564	0.584	0	2.902	0	6	6.295	3.919
methoxybenzene	2.633	-34.155	6	0	4.914	1.662	0	4.914	0	0	0	0	8	5.816	3.194
octane	3.694	-41.885	0	0	0	4.512	0	0	0	0	8.488	0	8	9.101	3.62
2-hexanone	2.792	-34.964	0	0	0	3.721	0	10.203	0	0	2.936	0	7	6.342	3.649
hexanol	2.964	-37.172	0	8.293	0	2.164	0	8.293	0	0	5.043	0.688	7	8.14	3.308
butanal	1.901	-26.083	0	0	0	1.981	0	9.405	0.594	0	1.684	0	5	6.296	3.931
propane	1.5	-16.448	0	0	0	4.25	0	0	0	0	1.25	0	3	9.915	3.93
butanol	2.085	-26.201	0	8.066	0	2.052	0	8.066	0	0	2.382	0.736	5	8.186	3.314
acetophenone	2.939	-39.627	6	0	0	1.565	0	10.645	0	0	0	0	9	5.212	4.36
toluene	2.525	-30.783	6	0	0	2.083	0	0	0	0	0	0	7	6.37	3.449
propylamine	1.747	-21.239	0	0	0	2.056	5.028	5.028	0	0	1.917	0.488	4	7.273	2.793
ethylbenzene	2.965	-35.913	6	0	0	2.162	0	0	0	0	1.14	0	8	6.397	3.442
propanol	1.645	-20.786	0	7.875	0	1.931	0	7.875	0	0	1.194	0.776	4	8.227	3.324
heptane	3.259	-36.807	0	0	0	4.491	0	0	0	0	7.009	0	7	9.224	3.67
nonanol	4.283	-54.05	0	8.469	0	2.23	0	8.469	0	0	9.301	0.65	10	8.118	3.302

Table S6. Continued

Molecule	AE	QM	nArom Bond	SsOH	SssO	SsCH3	SsNH2	SHBa	SHdsCH	nAcid	SssCH2	SHBd	nHeavy Atom	η	χ
octanol	3.843	-48.027	0	8.423	0	2.214	0	8.423	0	0	7.863	0.66	9	8.122	3.304
acetone	1.474	-19.875	0	0	0	3.056	0	9.444	0	0	0	0	4	6.358	3.719
butane	1.939	-21.54	0	0	0	4.361	0	0	0	0	2.639	0	4	9.759	3.863
hexane	2.819	-31.719	0	0	0	4.464	0	0	0	0	5.536	0	6	9.374	3.723
decane	4.577	-52.099	0	0	0	4.54	0	0	0	0	11.46	0	10	8.94	3.566
benzaldehyde	2.489	-35.728	6	0	0	0	0	10.006	0.7	0	0	0	8	5.182	4.593
pentylamine	2.626	-32.399	0	0	0	2.176	5.215	5.215	0	0	4.609	0.454	6	7.221	2.791
butanoic acid	2.09	-27.435	0	7.913	0	1.841	0	17.513	0	1	1.023	0.827	6	7.796	3.849
hexylamine	3.066	-37.502	0	0	0	2.205	5.269	5.269	0	0	6.026	0.444	7	7.204	2.793
methane	0.627	-6.325	0	0	0	0	0	0	0	0	0	0	1	12.052	4.646
pentanoic acid	2.53	-33.279	0	8.045	0	1.975	0	17.803	0	1	2.081	0.799	7	7.792	3.84
methanoic acid	0.759	-13.187	0	6.889	0	0	0	15.25	0.888	1	0	1.04	3	7.844	4.312
ethane	1.061	-11.368	0	0	0	4	0	0	0	0	0	0	2	10.457	4.088
nonane	4.138	-46.998	0	0	0	4.527	0	0	0	0	9.973	0	9	9.014	3.594
propanoic acid	1.643	-24.163	0	7.722	0	1.6	0	17.09	0	1	0.222	0.867	5	7.717	3.837
butylamine	2.187	-26.751	0	0	0	2.132	5.139	5.139	0	0	3.229	0.468	5	7.238	2.792
pentanol	2.524	-31.384	0	8.197	0	2.121	0	8.197	0	0	3.682	0.709	6	8.163	3.31
pentane	2.379	-26.637	0	0	0	4.424	0	0	0	0	4.076	0	5	9.571	3.798

Table S6. Continued

Molecule	AE	QM	nArom Bond	SsOH	SssO	SsCH3	SsNH2	SHBa	SHdsCH	nAcid	SssCH2	SHBd	nHeavy Atom	η	χ
2-heptanone	3.232	-39.58	0	0	0	3.787	0	10.319	0	0	4.243	0	8	6.339	3.643
ethanol	1.206	-15.433	0	7.569	0	1.681	0	7.569	0	0	0.25	0.839	3	8.254	3.375
heptanol	3.403	-42.649	0	8.366	0	2.193	0	8.366	0	0	6.441	0.673	8	8.13	3.305
heptanoic acid	3.409	-42.304	0	8.213	0	2.112	0	18.175	0	1	4.554	0.763	9	7.788	3.83
propanal	1.462	-19.883	0	0	0	1.813	0	9.174	0.61	0	0.639	0	4	6.29	3.911
ethanoic acid	1.211	-18.014	0	7.417	0	1.083	0	16.417	0	1	0	0.929	4	7.787	3.905
nonanoic acid	4.289	-54.488	0	8.316	0	2.178	0	18.404	0	1	7.251	0.741	11	7.801	3.867
hexanal	2.781	-35.383	0	0	0	2.13	0	9.68	0.577	0	4.209	0	7	6.32	3.956
butanone	1.913	-24.178	0	0	0	3.433	0	9.813	0	0	0.667	0	5	6.365	3.672
octanoic acid	3.849	-48.028	0	8.27	0	2.15	0	18.302	0	1	5.885	0.751	10	7.786	3.828
2-pentanone	2.353	-28.749	0	0	0	3.616	0	10.044	0	0	1.718	0	6	6.344	3.657
methanol	0.762	-9.921	0	7	0	1	0	7	0	0	0	0.95	2	8.32	3.392
butylbenzene	3.844	-64.129	6	0	0	2.225	0	0	0	0	3.83	0	10	6.376	3.417
methylamine	0.865	-10.554	0	0	0	1.5	4.5	4.5	0	0	0	0.575	2	7.335	2.792
benzene	2.081	-25.823	6	0	0	0	0	0	0	0	0	0	6	6.665	3.636
hexanoic acid	2.969	-37.903	0	8.14	0	2.057	0	18.014	0	1	3.277	0.778	8	7.789	3.834
benzyl_methyl_ether	3.066	-38.630	6	0	4.928	1.701	0	4.928	2.289	0	0.709	0	9	6.354	3.606

Table S6. Continued

Molecule	AE	QM	nArom Bond	SsOH	SssO	SsCH3	SsNH2	SHBa	SHdsCH	nAcid	SssCH2	SHBd	nHeavy Atom	η	χ
phenetole	3.088	-39.308	6	0	5.206	1.979	0	5.206	2.443	0	0.740	0	9	5.769	3.140
chloromethane	0.587	-15.321	0	0	0	2.164	0	1.182	0	0	0.000	0	2	8.512	3.819
chlorobuthane	1.903	-30.863	0	0	0	2.238	0	1.254	0	0	4.354	0	5	8.486	3.645
chloropentane	2.339	-37.605	0	0	0	2.250	0	1.256	0	0	5.840	0	6	8.260	3.741
chlorohexane	2.778	-43.393	0	0	0	2.259	0	1.256	0	0	7.330	0	7	8.371	3.675
chloropropane	1.466	-25.698	0	0	0	2.221	0	1.249	0	0	2.875	0	4	8.508	3.669
acetonitrile	0.934	-15.325	0	0	0	1.431	0	0	0	0	0.000	0	3	9.690	4.295
butyronitrile	1.808	-26.135	0	0	0	1.989	0	0	0	0	1.677	0	5	9.209	4.333
pentanenitrile	2.246	-31.725	0	0	0	2.081	0	0	0	0	2.903	0	6	9.147	4.268
hexanenitrile	2.683	-39.791	0	0	0	2.136	0	0	0	0	4.218	0	7	9.159	4.206
heptanonitrile	3.120	-45.832	0	0	0	2.173	0	0	0	0	5.585	0	8	9.074	4.159

Table S7. Complete dataset of polymer repeating elements, AE: atomization energy, QM: quadrupole moment, nAromBond: number of aromatic bonds, nHeavyAtom: number of heavy atoms (all but hydrogen), SsOH: sum of (-OH) E-States, SssO: sum of (-O-) E-States, nAcid: number of acidic groups, SHdsCH: sum of (=CH-) E-States, SsNH2: sum of (-NH2) E-States, SsCH3: sum of (-CH3) E-States, SHBa: sum of E-States for hydrogen bond acceptors, SHBd: sum of E-States for hydrogen bond donors, SssCH2: sum of (-CH2) E-States (see Supplementary Materials), η: chemical hardness, χ: electronegativity

RE	AE	QM	nArom Bond	SsOH	SssO	SsCH3	SsNH2	SHBa	SHdsCH	nAcid	SssCH2	SHBd	nHeavy Atom	η	χ
propanoic acid	1.643	-24.163	0	7.722	0	1.6	0	17.09	0	1	0.222	0.867	5	7.717	3.837
butanenitrile	1.816	-26.114	0	0	0	1.989	0	0	0	0	1.677	0	5	9.211	4.333
propanenitrile	1.376	-21.025	0	0	0	1.819	0	0	0	0	0.625	0	4	9.247	4.371
butane	1.939	-21.485	0	0	0	4.361	0	0	0	0	2.639	0	4	9.731	3.875
ethane	1.061	-11.369	0	0	0	4	0	0	0	0	0	0	2	10.457	4.088
dimethyl ether	1.19	-14.873	0	0	4.25	3.25	0	4.25	0	0	0	0	3	8.207	2.987
2-methyl-2-butene	2.174	-24.944	0	0	0	6.204	0	0	0.332	0	0	0	5	6.55	2.921
isobutane	1.942	-21.581	0	0	0	6.5	0	0	0	0	0	0	4	9.619	3.916
2-methylpropanenitrile	1.817	-26.237	0	0	0	3.722	0	0	0	0	0	0	5	9.174	4.316
methyl butyrate	2.505	-31.452	0	0	4.352	3.342	0	14.541	0	0	1.406	0	7	7.668	3.63
propane	1.5	-16.448	0	0	0	4.25	0	0	0	0	1.25	0	3	9.915	3.93
ethylbenzene	2.965	-35.913	6	0	0	2.162	0	0	0	0	1.14	0	8	6.397	3.442
ethanol	1.206	-15.319	0	7.569	0	1.681	0	7.569	0	0	0.25	0.839	3	8.254	3.375
ethyl acetate	2.082	-27.151	0	0	4.403	3.172	0	14.224	0	0	0.481	0	6	7.737	3.686
chloroethane	1.032	-20.641	0	0	0	2.198	0	1.234	0	0	1.414	0	3	8.516	3.702
diethyl ether	2.079	-24.492	0	0	4.833	3.979	0	4.833	0	0	1.688	0	5	8.03	2.973

S3 Reference

1. Hall, L.H.; Kier, L.B. Electrotopological State Indexes for Atom Types - a Novel Combination of Electronic, Topological, and Valence State Information. *J. Chem. Inf. Comp. Sci.* **1995**, *35*, 1039-1045.
2. William E. Acree, J.; Chickos, J.S. Phase Transition Enthalpy Measurements of Organic and Organometallic Compounds. In *NIST Chemistry WebBook, NIST Standard Reference Database Number 69*, P.J. Linstrom, Mallard, W.G., Eds.; National Institute of Standards and Technology: Gaithersburg MD, 20899, 2021.
3. Lide, D.R. *CRC handbook of chemistry and physics*, 97 ed.; CRC press: 2016.
4. Verevkin, S.P. Measurement and prediction of the monocarboxylic acids thermochemical properties. *J. Chem. Eng. Data* **2000**, *45*, 953-960.
5. Chemical Retrieval on the Web (CROW). Available online: http://www.polymerdatabase.com/ (accessed on 24.10.2021).
6. Barton, A.F. *Handbook of polymer-liquid interaction parameters and solubility parameters*; Routledge: 2018.
